# Validation of Housekeeping Genes in the Brains of Rats Submitted to Chronic Intermittent Hypoxia, a Sleep Apnea Model

**DOI:** 10.1371/journal.pone.0109902

**Published:** 2014-10-07

**Authors:** Guilherme Silva Julian, Renato Watanabe de Oliveira, Juliana Cini Perry, Sergio Tufik, Jair Ribeiro Chagas

**Affiliations:** 1 Departamento de Psicobiologia, Universidade Federal de São Paulo, (UNIFESP), São Paulo, São Paulo, Brazil; 2 Departamento de Biociências, Universidade Federal de São Paulo (UNIFESP-Baixada Santista), Santos, São Paulo, Brazil; Nathan Kline Institute and New York University School of Medicine, United States of America

## Abstract

Obstructive sleep apnea (OSA) is a syndrome characterized by intermittent nocturnal hypoxia, sleep fragmentation, hypercapnia and respiratory effort, and it has been associated with several complications, such as diabetes, hypertension and obesity. Quantitative real-time PCR has been performed in previous OSA-related studies; however, these studies were not validated using proper reference genes. We have examined the effects of chronic intermittent hypoxia (CIH), which is an experimental model mainly of cardiovascular consequences of OSA, on reference genes, including *beta-actin, beta-2-microglobulin, glyceraldehyde-3-phosphate dehydrogenase, hypoxanthine guanine phosphoribosyl transferase and eukaryotic 18S rRNA,* in different areas of the brain. All stability analyses were performed using the geNorm, Normfinder and BestKeeper software programs. With exception of the *18S rRNA*, all of the evaluated genes were shown to be stable following CIH exposure. However, gene stability rankings were dependent on the area of the brain that was analyzed and varied according to the software that was used. This study demonstrated that CIH affects various brain structures differently. With the exception of the *18S rRNA,* all of the tested genes are suitable for use as housekeeping genes in expression analyses.

## Introduction

Obstructive sleep apnea (OSA) is the most common sleep-related breathing disorder. OSA has been associated with several comorbidities, such as cardiovascular disease, hypertension, diabetes, cognitive impairments and metabolic syndrome [1]–[7] **.** It has been recently estimated to affect approximately one-third of the population of São Paulo, Brazil [8]. OSA is characterized by the recurrent closure or partial collapse of the upper airway, resulting in hypercapnia, increased respiratory efforts, sleep fragmentation and intermittent hypoxia (IH). Hypoxia seems to be one of the most important components of OSA. Many animal models that are commonly been used in the study of hypoxia have been developed over the years, of which the most widely used is the chronic intermittent hypoxia (CIH) model, which simulates only one factor of OSA. CIH, occurring isolated or in association with sleep fragmentation, has been demonstrated to lead to several changes that are similar to those found in individuals with OSA, such as cognitive impairment, insulin resistance and hypertension [1], [9]–[12]. In fact, CIH is responsible for the activation of the sympathetic nervous system, leading to the development of hypertension [13]. Recently, it has been demonstrated that CIH acts by modulating presympathetic neurons activity in the rostral ventrolateral medulla [14], increasing sympathetic activity. These data suggest that intermittent hypoxia plays an important role in OSA. Although CIH model lacks of several OSA factors, it is still an important tool to study OSA, mainly in cardiovascular area.

Real-time polymerase chain reaction (RT-PCR) is a method that allows for the measurement of the gene expression of specific targets [15] to better understand biological processes. Due to its efficiency, RT-PCR has rapidly become a well-established technique that is widely used for the quantification of mRNA levels using a technique called quantitative RT-PCR (qRT-PCR). Normally, qRT-PCR is based on relative quantifications that consist of normalizing the target gene with an internal control, which is a gene that presumably maintains stable expression during the experiment and is termed a housekeeping gene (HKG) [16]. HKGs have been validated for several experimental models and tissues; however, proper precautions are not always taken to account for their variabilities, thereby compromising the reliability of the data [17]–. There are several possible HKGs that may be utilized, the most common of which are *glyceraldehyde-3-phosphate dehydrogenase (GAPDH)* and *beta-actin* (*ACTB)*. Although their uses have been well-established, they have been demonstrated to possess low stability in an *in vitro* hypoxia model [20].

Our group has previously validated the optimal HKGs for use with other sleep impairment models [21], but the effects of sleep-related breathing disorders, such as OSA, on HKGs have not yet been studied. Considering the increasing number of studies involving CIH models, including those using genetic approaches [22], [23], and particularly qRT-PCR, we aimed to validate HKGs for use in studies involving the commonly used CIH model.

## Materials and Methods

### Animals and ethics statement

Male adult Wistar Hannover rats (n=8 *per* group) that were provided by the Centro de Desenvolvimento de Modelos Experimentais para Medicina e Biologia (CEDEME) at the Universidade Federal de São Paulo were submitted for use in the experimental CIH model. The animals were housed in a room at 22°C with a  12: 12 h light-dark cycle (lights on at 7:00) and allowed access to food and water *ad libitum*. All of the experimental procedures were performed according to the ethical and practical guidelines for the use of laboratory animals [24]. All of the procedures that were used in the present study complied with the National Institutes of Health Guide for the Care and Use of Laboratory Animals. This study was approved by the Animal Experimental Ethics Committee of the Universidade Federal de São Paulo (#2025/11).

### Chronic intermittent hypoxia

**Figure 1:**
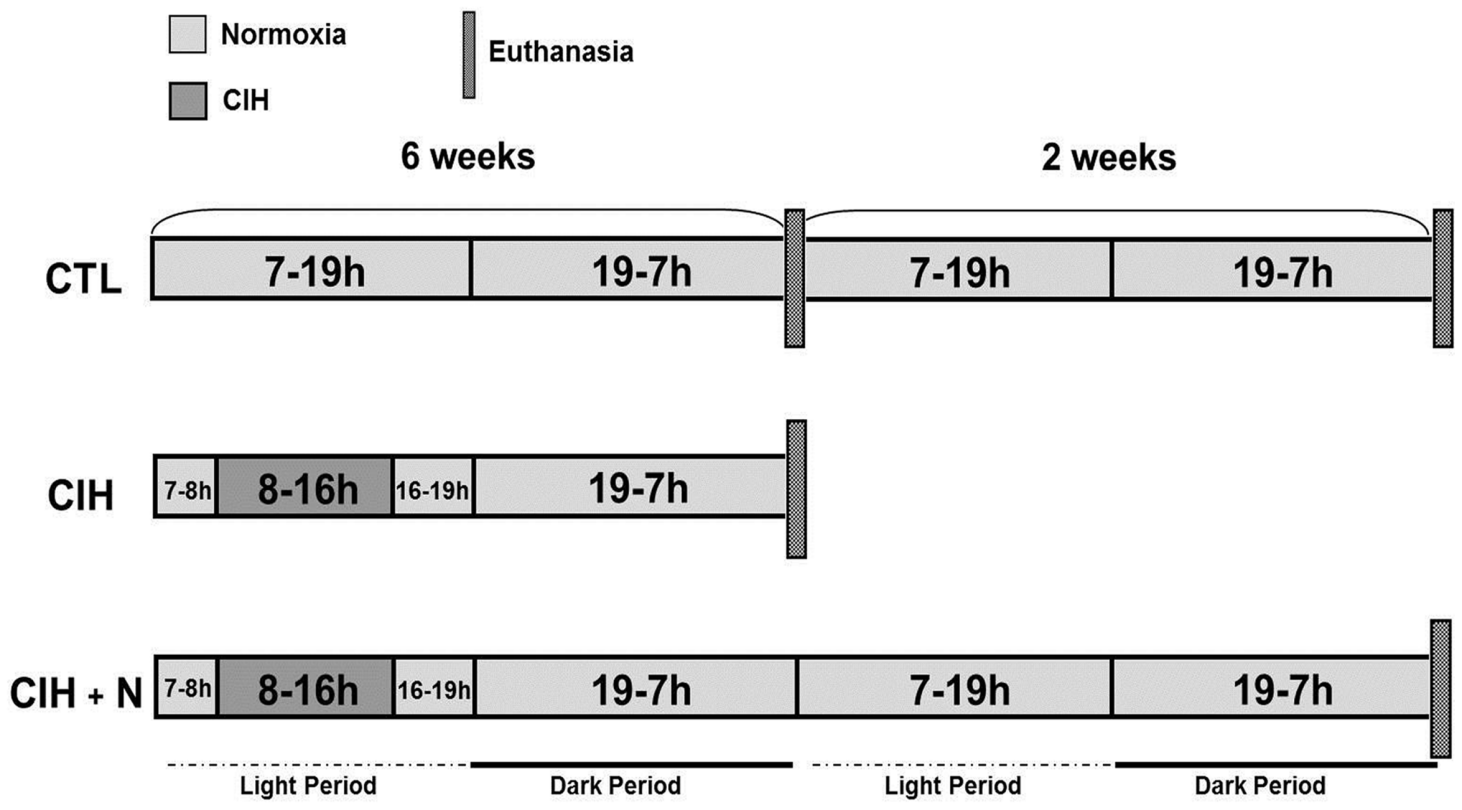
Experimental protocol. Experimental timeline for each group: CTL (control group, n=8); CIH (animals subjected to 8-hours/day of chronic intermittent hypoxia for six weeks, n=8); CIH+N (animals subjected to 8-hours/day of chronic intermittent hypoxia for six weeks followed by a two-week recovery period, n=8).

The IH procedure was induced in a commercially designed chamber (30 in×20 in×20 in Oxycycler model A44XOV, Biospherix, Redfield, NY, USA) that was connected to O_2_ and N_2_ supplies. The O_2_ concentration was controlled in real time by an O_2_ analyzer coupled to a computerized system that controlled the O_2_ and N_2_ outlets. The O_2_ concentrations were adjusted by the addition of N_2_ or O_2_. The chamber was maintained with ambient CO_2_ levels of <0.02 ppm, humidity levels of between 40–50% and temperatures of 22–24°°C. The IH consisted of repeated cycles with 3-minute ramp (from 5–21% and from 21–5%), varying O_2_ concentrations ranging from 5–21% for  8 h a day during the light period. TIt has previously been demonstrated that exposure to IH at a nadir O_2_ concentration of 10% is capable of inducing the desaturation of oxyhemoglobin to levels of 40% [25]. The rats were randomly assigned to 3 experimental groups containing 8 animals each, as follows: 1) the control group (CTL); 2) the CIH group (CIH), which was subjected to IH for six weeks; and 3) the CIH +2 weeks of normoxia group (CIH+N), which was subjected to the CIH protocol followed by 2 weeks of recovery in normoxia [26] (Fig. 1).

### Housekeeping gene selection

The most commonly used genes in the literature were evaluated in our CIH model. Because CIH affects several systems, different gene pathways were chosen to ensure for increased coverage. The genes that were selected included eukaryotic *18S rRNA* (*18S*), *ACTB*, *beta-2-microglobulin* (*β2M*), *GAPDH* and *hypoxanthine guanine phosphoribosyl transferase* (*HPRT*). *18S* plays an essential role in maintaining ribosomal structures, *ACTB* plays a structural role in the cytoskeleton, *β2M* participates in MHC type I antigen presentation, *GAPDH* participates in glycolysis by converting glyceraldeyde-3-phosphate to D-glycerate-1,3-biphosphate, and *HPRT* converts hypoxanthine to inosine monophosphate, thus playing a role in purine generation (Table 1).

**Table 1:** Candidate genes, accession numbers and primer sequences.

Gene symbol and accession number	Gene name	Primer sequence	Product length (bp)
***GAPDH***	*Glyceraldehyde-3-phosphate*	F 5′ -TGCCCCCATGTTTGTGATG- 3′	64
**NM_017008**	*dehydrogenase*	R 5′ -GCTGACAATCTTGAGGGAGTTGT- 3′	
***ACTB***	*Beta-actin*	F 5′ -AGCGTGGCTACAGCTTCACC- 3′	85
**NM_031144**		R 5′ -AAGTCTAGGGCAACATAGCACAGC- 3′	
***β2M***	*Beta-2-microglobulin*	F 5′ -GCCATCCACCGGAGAATG- 3′	60
**NM_012512**		R 5′ -GGTGGAACTGAGACACGTAGCA- 3′	
***HPRT***	*Hypoxanthine guanine*	F 5′ -GCGAAAGTGGAAAAGCCAAGT- 3′	76
**NM_012583**	*phosphoribosyl transferase*	R 5′ -GCCACATCAACAGGACTCTTGTA- 3′	
***18S***	*Eukaryotic 18S rRNA*	F 5′ -CGGACAGGATTGACAGATTG- 3′	83
**NR_046237**		R 5′ -CAAATCGCTCCACCAACTAA- 3′	

### RNA extraction, quantification and reverse transcription

The animals were euthanized following the CIH and normoxia recovery protocols by rapid decapitation without anesthetic treatment, due to possible interference in HKG expression [27]. Next, the brain was rapidly removed, and the hippocampus, hypothalamus, and frontal and temporal cortices were dissected, using a scalpel, in a Petri plate in dry ice. All of the tissues were rapidly dissected on dry ice and kept at −−80°°C until extraction. Total RNA extraction was performed for all of the structures using TRIzol (Invitrogen, CA, USA) according to the manufacturer’s instructions. The RNA samples were then treated with DNase I (Invitrogen), and their qualities and integrities were assessed by rRNA visualization using agarose gel electrophoresis. Quantitation was performed using spectrophotometry at 260 nm (NanoDrop), and purity was estimated according to an optimal 260/280 nm ratio of 1",0,0,2$10#?>>1.8. One microgram of RNA from each dissected structure was reverse transcribed using the High Capacity cDNA Reverse Transcription Kit (Applied Biosystems) according to the manufacturer’s instructions.

### qRT-PCR

Each cDNA was used as a template for real-time PCR (RT-PCR) amplification using SYBER green (Applied Biosystems) and the Applied Biosystems 7500 Real-Time PCR System (Applied Biosystems). Each reaction was performed in a final volume of 20 µl, containing 1 µL of cDNA that was diluted with H_2_O and 19 µL of master mix (10 µL of SYBER green and 9 µL of water containing reverse and forward primers at a 90 nM final concentration). The Ct values were maintained between 15.0 and 33.0. The expression level of each HKG was assessed using the 2^−ΔCt^ equation [28]. All samples were run in triplicate, and average values were calculated.

### Data analyses

Gene expression stability was evaluated using three of the most common relevant methods, including geNorm [28], NormFinder [29] and BestKeeper [30]. The 2^−ΔCt^ value of each gene was used to evaluate stability for all of the methods. geNorm calculates an M-value, which describes the variation of the expression of a gene compared with all of the other candidate genes; a lower M-value indicates a more stable gene [28]. Normfinder estimates the changes in the candidate HKG expression, considering the gene and experimental conditions, and computes a stability value number; more stable genes possess lower stability values [29]. BestKeeper provides an HKG index using a pairwise correlation and calculates a p Corr value, which is a P value of a Pearson correlation; values that are closer to 1.0 suggest more stable HKGs [30]. geNorm [31], NormFinder [32] and BestKeeper [33] were downloaded for free.

### Statistical analyses

All of the data were independently analyzed using the 2^−ΔCt^ equation to ensure the absence of any variation between groups [34]. For normality and homogeneity, the Kolmogorov-Smirnov test and Levene’s test were performed, respectively. If necessary, a z-score correction was performed, and a group comparison was conducted using one-way ANOVA. Statistical significance was set at P<0.05.

## Results

### RNA quality

RNA quality is a crucial issue when studying gene expression. For this reason, all of the samples were initially treated with DNase I, and their integrity was evaluated by the assessment of intact 28S and 18S rRNAs on agarose gels.

### qRT-PCR

Pilot assays were performed to optimize the cDNA and primer quantities. A total of 0.9 µg of RNA that was previously treated with DNase was used for the reverse transcription reaction in a total volume of 40 µl. One microliter of the resulting cDNA was used for the qPCR reaction. Each gene amplification was analyzed, and a melting curve analysis was performed, showing a single peak indicating the temperature of dissociation. Ct values were maintained between 15.0 and 33.0, and all procedures were adapted from Lee et al. [21]. All of the 2^−ΔCt^ values were independently analyzed by a one-way ANOVA, which indicated that the candidate genes were not significantly altered. All comparisons were performed at a significance of 0",0,0,2$10#?>P>0.05 (Data not shown).

### geNorm analysis

To identify the optimal HKGs for use in the study of CIH, a geNorm analysis was performed of all five candidate genes for each of the four brain structures. Additionally, the same analysis was performed for all of the structures collectively. All of the candidate genes in all of the analyses presented with M-values of lower than 1.5, which is considered to be the cut-off for suitability. The analysis of the hippocampus, hypothalamus, and frontal and temporal cortices together as one single area resulted in increased M-values for all candidates due to the inter-structural variabilities; however, these values still remained lower than 1.5.

**Figure 2:**
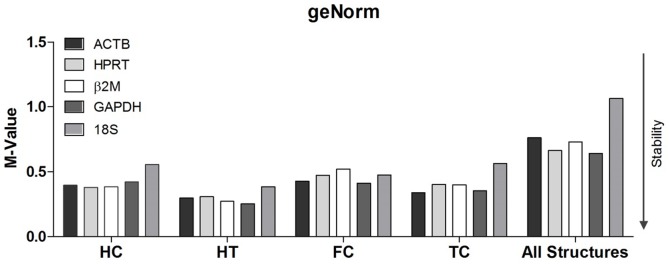
geNorm stability analysis. Evaluation of gene expression stability by geNorm for individual brain regions. All HKG candidates presented values that were lower than the cut-off value (1.5). HC, hippocampus; HT, hypothalamus; FC, frontal cortex; TC, temporal cortex.

For the temporal cortex, the geNorm evaluation determined that the best candidate was *ACTB* followed by *GAPDH*. Additionally, the least stable gene for this structure was *18S*, which presented with the highest M-value. For the frontal cortex, the candidate gene rank was similar to that of the temporal cortex, with *GAPDH* and *ACTB* possessing the lowest M-values and thus being the best pair of candidates and *GAPDH* being the most stable followed by *ACTB*. The least stable gene with the highest M-value was *β2M*. For the hippocampus, the geNorm analysis determined that the best pair of candidates was *HPRT* and *ACTB,* and the least stable was *18S*. The analysis of the hypothalamus revealed the most stable values for all genes, and the best pair of candidates was *GAPDH* and *β2M*. The least stable gene was *18S*, which was the least stable for all of the regions except the frontal cortex. The analysis of all of the brain regions together indicated that the more stable pair of candidates was *GAPDH* and *HPRT*, and the least stable was *18S*, which presented with an M-value of 1.06 and was the closest value to the cut-off of 1.5. All of the M-values are presented in Table 2, and data from all of the brain structures are shown in Fig. 2.

**Table 2:** Candidate HKGs in geNorm evaluation.

Rank	Temporal Cortex		Frontal Cortex		Hippocampus		Hypothalamus		Total	
	Gene	M-value	Gene	M-value	Gene	M-value	Gene	M-value	Gene	M-value
**1**	*ACTB*	0.338	*GAPDH*	0.411	*HPRT*	0.378	*GAPDH*	0.252	*GAPDH*	0.642
**2**	*GAPDH*	0.353	*ACTB*	0.427	*β2M*	0.384	*β2M*	0.271	*HPRT*	0.663
**3**	*β2M*	0.399	*HPRT*	0.473	*ACTB*	0.395	*ACTB*	0.296	*β2M*	0.728
**4**	*HPRT*	0.402	*18S*	0.474	*GAPDH*	0.420	*HPRT*	0.308	*ACTB*	0.762
**5**	*18S*	0.563	*β2M*	0.520	*18S*	0.555	*18S*	0.383	*18S*	1.065

Ranking of candidate HKGs by geNorm for each brain structure and all structures together as described by the total M-value. Lower M-values indicate more stable genes. A cut-off value of 1.5 was used.

### NormFinder analysis

NormFinder analyses were performed for all five candidate genes and for each brain structure and also for all of the structures together. All candidate genes in all brain regions presented with stability values of lower than 0.15, which was the cut-off for suitability [28]. The collective analysis of the hippocampus, hypothalamus, and frontal and temporal cortices led to increased stability values for all of the candidates due to the inter-structural variabilities; however, they remained under the cut-off value of 0.15. These results were very similar to those of geNorm for all structures.

**Figure 3:**
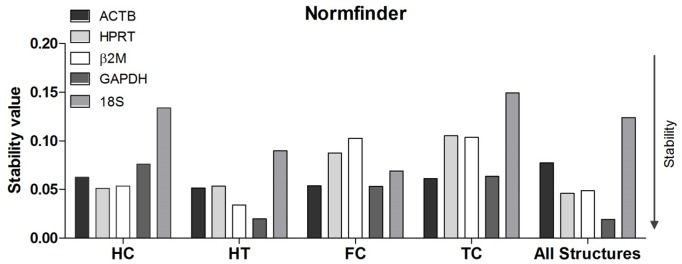
Normfinder stability analysis. Evaluation of gene expression stability by Normfinder for individual brain regions. All HKG candidates presented values that were lower than the cut-off value (0.15). HC, hippocampus; HT, hypothalamus; FC, frontal cortex; TC, temporal cortex.

For the temporal cortex, the results that were obtained using NormFinder were the same as those from geNorm, which showed that *ACTB* and *GAPDH* were the best pair of candidates and that *18S* was the least stable. The analysis of the frontal cortex revealed nearly the same ranking as that of geNorm; the best pair of candidates was *GAPDH* and *ACTB*, and the least stable was *Β2M,* which was consistent in both analyses. For the hippocampus, the results were slightly different from those of geNorm; the best pair of candidates was *HPRT* and *β2M* (Table 3), and the least stable was *18S*, which remained the same for both analyses, presenting with a stability value of 0.134, which was the closest to the cut-off of 0.15. For the hypothalamus, the NormFinder analysis presented with the same results as those of geNorm, with the best pair of candidates being *GAPDH* and *β2M* and *18S* being the least stable. The collective analysis of all of the brain structures indicated that *GAPDH* and *HPRT* were the best candidates and that *18S* was the least stable (Fig. 3). The stability value for *18S* was 0.124, which was very close to the 0.15 cut-off value.

**Table 3:** Candidate HKGs in NormFinder evaluation.

Rank	Temporal Cortex		Frontal Cortex		Hippocampus		Hypothalamus		Total	
	Gene	Stab. Value	Gene	Stab. Value	Gene	Stab. Value	Gene	Stab. Value	Gene	Stab. Value
**1**	*ACTB*	0.061	*GAPDH*	0.053	*HPRT*	0.051	*GAPDH*	0.019	*GAPDH*	0.019
**2**	*GAPDH*	0.063	*ACTB*	0.053	*β2M*	0.053	*β2M*	0.034	*HPRT*	0.045
**3**	*β2M*	0.103	*18S*	0.069	*ACTB*	0.062	*ACTB*	0.051	*β2M*	0.048
**4**	*HPRT*	0.105	*HPRT*	0.087	*GAPDH*	0.076	*HPRT*	0.053	*ACTB*	0.077
**5**	*18S*	0.149	*β2M*	0.102	*18S*	0.133	*18S*	0.089	*18S*	0.124

Ranking of candidate HKGs by NormFinder for each brain structure and all structures together, as described by the total M-value. Lower stability values indicate more stable genes. A cut-off value of 0.15 was used.

### BestKeeper analysis

BestKeeper analyses were performed for all five candidate genes and each brain structure as well as all four structures collectively. All of the candidate genes presented with acceptable values, with the exception of *18S* in the temporal cortex, which possessed a non-significant *P* value (≤P≤0.05), as determined by the Pearson correlation. The results that were obtained from the BestKeeper software differed from those of NormFinder and geNorm for most of the genes. All p Corr values are listed in Table 4.

**Table 4:** Candidate HKGs in BestKeeper evaluation.

Rank	Temporal Cortex		Frontal Cortex		Hippocampus		Hypothalamus		Total	
	Gene	R	Gene	R	Gene	R	Gene	R	Gene	R
**1**	*HPRT*	0.94	*ACTB*	0.889	*ACTB*	0.926	*ACTB*	0.954	*GAPDH*	0.853
**2**	*β2M*	0.912	*GAPDH*	0.859	*HPRT*	0.923	*GAPDH*	0.943	*β2M*	0.677
**3**	*ACTB*	0.905	*β2M*	0.754	*GAPDH*	0.831	*β2M*	0.901	*HPRT*	0.676
**4**	*GAPDH*	0.882	*HPRT*	0.623	*β2M*	0.81	*HPRT*	0.896	*18S*	0.585
**5**	18S*	0.199	*18S*	0.544	*18S*	0.615	*18S*	0.67	*ACTB*	0.558

Ranking of candidate HKGs by BestKeeper for each brain structure and all structures together, as described by total. Higher correlation coefficients (R) indicate more stable genes. All values achieved a significance of *P*<0.05, according to Pearson correlation analysis, with the exception of 18S in the temporal cortex. A cut-off value of P>0.05 was used for Pearson correlation analysis was set. *:P>0.05.

**Figure 4:**
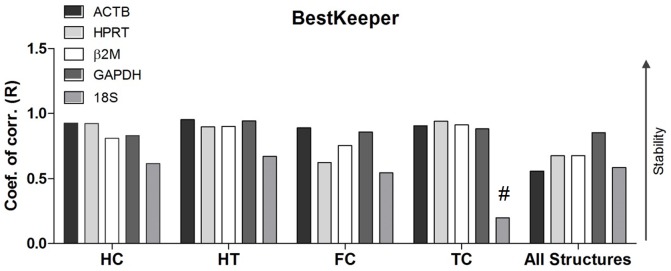
BestKeeper stability analysis. Evaluation of gene expression stability by BestKeeper for individual brain regions. The Pearson correlation coefficient (R) of all HKG candidates. All HKG except 18S in the temporal cortex, presented significant correlation (P<0.05), as determined by Pearson correlation analysis. #: P>0.05 HC, hippocampus; HT, hypothalamus; FC, frontal cortex; TC, temporal cortex.

For the temporal cortex, in contrast to the findings that were obtained from geNorm and NormFinder, the best pair of candidates was found to be *HPRT* and *β2M*; however, *18S* remained the least stable, presenting with a *P* value of 0.35, as determined by the Pearson correlation analysis. These results suggest that it is not a suitable HKG for use in gene expression studies. The analysis of the frontal cortex indicated that the pair of best candidates was the same as that revealed by geNorm and NormFinder. However, in contrast to the other two analyses, the least stable gene was observed to be *18S*. For the hippocampus, the candidate genes were the same as those reported by the other methods; however, *ACTB* was followed by *HPRT* according to the BestKeeper software. The least stable candidate was also determined to be *18S*. For the hypothalamus, the pair of best candidates was *ACTB* and *GAPDH*. In this case, *GAPDH* remained as one of the best candidates, in accordance with findings noted previously, and the least stable candidate was *18S*, which is in agreement with the findings observed in the other structures. The collective analysis of all of the structures by BestKeeper showed that *GAPDH* and *β2M* were the best pair of candidate genes, which slightly differed from the results reported by geNorm and NormFinder; however, *18S* remained as the least stable gene (Fig. 4).

## Discussion

qRT-PCR is the most commonly used method for the quantification of mRNA; however, its reliability depends on the correct normalization of the results using stable HKGs, a bad choice of which can compromise the results [19].

There have been several studies involving HKGs that have been performed using different hypoxia models [17], [19], [20], but most of them have been conducted *in vitro* with an acute hypoxia model [20], [35]. These studies are usually conducted by subjecting a specific cell line to differing oxygen concentrations. In some of them, commonly used HKGs were observed to exhibit altered expression levels in hypoxic conditions [17].

The current study showed that all of the analysis software programs produced similar results, particularly geNorm and NormFinder. For the analysis using BestKeeper software, the only difference was observed in the temporal cortex, in which different optimal candidates were revealed compared with those identified by the other 2 software programs.

NormFinder and geNorm presented similar HKG ranks, the similarity may be due to pairwise comparison methods used in both [28], [36], for the same reason, BestKeeper presents different ranks, when compared to NormFinder and geNorm, due to comparison method by Pearson correlation (P value and correlation coefficient (R)) that is to rank HKG [30].

Our data indicate that all of the candidate genes that were tested are suitable for use according to the adopted cut-off values. The only exception is *18S*, which was the least stable gene in almost all of the structures, independent of the method of evaluation, in contrast with previous studies involving *in vitro* hypoxia [37], [38]. Thus, *18S* is not advisable as an HKG under any conditions, due to poor ranking position in most structures. Our data demonstrated that *18S* was not stable following CIH exposure, corroborating previous studies reporting that its expression varies according to the cell line that is being tested under hypoxic conditions. *18S* has been shown to be stable in HEK (embryonic kidney) and PNT2 (normal prostate epithelium) cells, but to also be the least stable in LNCap (prostate cancer) and MCF-7 (breast cancer) cells [37]. Interestingly, *18S* stability varies in the PNT2 and LNCap cells in a contrasting manner; both are present in the same tissue type but under different conditions; i.e., physiological and pathological conditions, respectively. A study of the brain of an *in vivo* model of CIH revealed that *18S* stability is homogeneous, demonstrating the sensitivity of a majority of brain cells to the CIH model in all structures.

No data are currently available in the literature describing HKGs in specific brain structures using hypoxia models, and most of the studies have been performed *in vitro.* In an acute hypoxia model using neural stem cells, commonly used HKGs, such as *ACTB* and *GAPDH,* showed altered expression levels [20]. Our data demonstrated that both *ACTB* and *GAPDH* were stable following CIH exposure and were the most stably expressed in the cortices. Additionally, our data correlate with those of a similar study of neural stem cells in acute hypoxia [20], which indicated that *HPRT* exhibits homogeneous gene expression. *HPRT* was also reported to be the most stable candidate in an analysis of the effects of CIH on the hippocampus, which is the main producer of stem cells in the adult brain [39].

In general, *β2M* was among the least stable candidate genes. The only exception was the hypothalamus, where it was one of the most stably expressed. *β2M* stability has been demonstrated to vary in a structure-dependent manner. It has been reported to be one of the most stable HKGs in cardiosphere-derived cells that have been subjected to acute hypoxia [35]. However, an analysis of MCF-7, PNT2, and LNCaP cell lines that were also subjected to acute hypoxia revealed that *β2M* was one of the least stable candidates [19], supporting our observations of its inter-structural variability in expression.

A large inter-structural variability between genes was observed. Each structure was shown to have a unique optimal HKG, demonstrating that the CIH model affects different cascades in a structure-dependent manner. Interestingly, the temporal and frontal cortices showed very similar rankings, which is possibly because both areas originated from the same embryonic brain vesicle, the telencephalon [40].

The evaluation of all of the structures together revealed increased variability and reduced stability. This large variability may be due to the structure-dependent differences in mRNA levels. In fact, the evaluation of all of the tested structures together can be useful when studying more than one structure.

As stated above this study presents some limitations: CIH model only simulates one of the four major factors of OSA; not all brain structures important for OSA or CIH model were studied. Additionally, only the structures with higher incidence of genetic studies were included. Nonetheless, the work has demonstrated that all of the tested candidate HKGs, but 18S, are suitable for use. Notably, the stability varied in a structure-dependent manner, and each structure possessed its own optimal HKG. The use of more than one HKG is strongly advisable to obtain reliable data.
